# Implementation of clinical decision support in young children with acute gastroenteritis: a randomized controlled trial at the emergency department

**DOI:** 10.1007/s00431-016-2819-2

**Published:** 2016-12-08

**Authors:** Dorien Geurts, Evelien de Vos-Kerkhof, Suzanne Polinder, Ewout Steyerberg, Johan van der Lei, Henriëtte Moll, Rianne Oostenbrink

**Affiliations:** 1 Department of Pediatrics, Erasmus MC - Sophia children’s hospital, Wytemaweg 80, Rotterdam, CN 3015 The Netherlands; 2 Department of Public Health, Erasmus MC, Rotterdam, The Netherlands; 3 Department of Medical Informatics, Erasmus MC, Rotterdam, The Netherlands

**Keywords:** Acute gastroenteritis, Children, Randomized control trial, Clinical decision support system, Emergency department

## Abstract

Acute gastroenteritis (AGE) is one of the most frequent reasons for young children to visit emergency departments (EDs). We aimed to evaluate (1) feasibility of a nurse-guided clinical decision support system for rehydration treatment in children with AGE and (2) the impact on diagnostics, treatment, and costs compared with usual care by attending physician. A randomized controlled trial was performed in 222 children, aged 1 month to 5 years at the ED of the Erasmus MC-Sophia Children’s hospital in The Netherlands ( 2010–2012). Outcome included (1) feasibility, measured by compliance of the nurses, and (2) length of stay (LOS) at the ED, the number of diagnostic tests, treatment, follow-up, and costs. Due to failure of post-ED weight measurement, we could not evaluate weight difference as measure for dehydration. Patient characteristics were comparable between the intervention (*N* = 113) and the usual care group (*N* = 109). Implementation of the clinical decision support system proved a high compliance rate. The standardized use of oral ORS (oral rehydration solution) significantly increased from 52 to 65%(RR2.2, 95%CI 1.09–4.31 *p* < 0.05). We observed no differences in other outcome measures.

*Conclusion*: Implementation of nurse-guided clinical decision support system on rehydration treatment in children with AGE showed high compliance and increase standardized use of ORS, without differences in other outcome measures.

**Table:** 

**What is Known:**
*• Acute gastroenteritis is one of the most frequently encountered problems in pediatric emergency departments.*
*• Guidelines advocate standardized oral treatment in children with mild to moderate dehydration, but appear to be applied infrequently in clinical practice.*
**What is New:**
*• Implementation of a nurse-guided clinical decision support system on treatment of AGE in young children showed good feasibility, resulting in a more standardized ORS use in children with mild to moderate dehydration, compared to usual care.*
*• Given the challenges to perform research in emergency care setting, the ED should be experienced and adequately equipped, especially during peak times.*

## Introduction

Acute gastroenteritis (AGE) is one of the most frequent reasons for young children to visit the emergency department (ED). Clinical dehydration scores are often used to assess severity of dehydration. These scores attempt to differentiate children without signs of dehydration from those with moderate dehydration or those with severe dehydration with signs of hypovolemic shock [10, 11]. The most commonly used clinical dehydration scale (CDS) is a 4-point scale, which includes four clinical signs (i.e., general appearance, eyes, mucous membranes, and tears) [10] The CDS has been incorporated in several clinical guidelines for appropriately managing acute gastroenteritis or dehydration [1, 13, 17, 25]. Although clinical guidelines aim to assist standardized assessment and treatment of dehydration, clinicians often do not adhere to the guidelines’ recommendations. Incorporating a guideline in an electronic, easily accessible clinical decision support system can improve guideline-adherence and therefore quality of care [18]

In this study, we aimed to evaluate the feasibility of an electronic, easily accessible, guideline-based clinical decision support system, as well as the impact of this nurse-guided clinical decision support system for managing children with AGE at the ED compared to usual care on diagnostics, treatment and costs.

## Methods

### Design

We conducted a randomized controlled trial comparing management of children with AGE at risk for dehydration by clinical decision support recommendations with usual care (Nederlands Trial Register (NTR), http://www.trialregister.nl/trialreg/index.asp; NTR2304).

### Patients and setting

We included children with acute vomiting and/ or diarrhea, aged 1 month to 5 years, who visited the ED of the Erasmus MC-Sophia, Rotterdam between May 2010 and December 2012. The Erasmus MC-Sophia is an inner-city pediatric university hospital, with annually 9000 children presenting at the ED [19]. About 35% of the ED population has chronic co-morbidity. [23] We excluded children with chronic diarrhea (>7 days), severe dehydration with hypovolemic shock, children with vomiting/diarrhea with a focus for another infectious disease (e.g., otitis media, urinary tract infection) and chronically ill children with complex needs.

Ethical approval for this study was obtained by the institutional review board (IRB) of the Erasmus MC. Informed consent was required and obtained from all parents (MEC-2008-071).

### Standard of care: initial patient assessment and treatment

Following the standard of care, during the process of triage, trained nurses registered vital signs and weight, as well as signs and symptoms and risk factors for dehydration for all patients [24].

Patients randomized to usual care were evaluated by the attending physician who subsequently decided on further rehydration management based on the patients clinical assessment and estimated level of dehydration. Our current guidelines advised rehydration treatment of 10–20 ml/kg/h during the length of stay at ED, followed by parental guidance on fluid maintenance as well as treatment of ongoing losses was advised [8] If oral rehydration did not succeed due to refusal of oral intake ór persistent vomiting, secondly, rehydration by a nasogastric tube was started. As rehydration therapy already was mostly based on oral rehydration using ORS, they received ORS or any rehydration fluid as prescribed by the attending physician. Anti-emetics were not a part of our national guideline, as evidence on the use of ant-emetics, as well as on safety of ondansetron, was lacking. The guideline could be retrieved from the protocol server of the Erasmus MC website on initiative of the clinician.

### The intervention

**Fig. 1:**
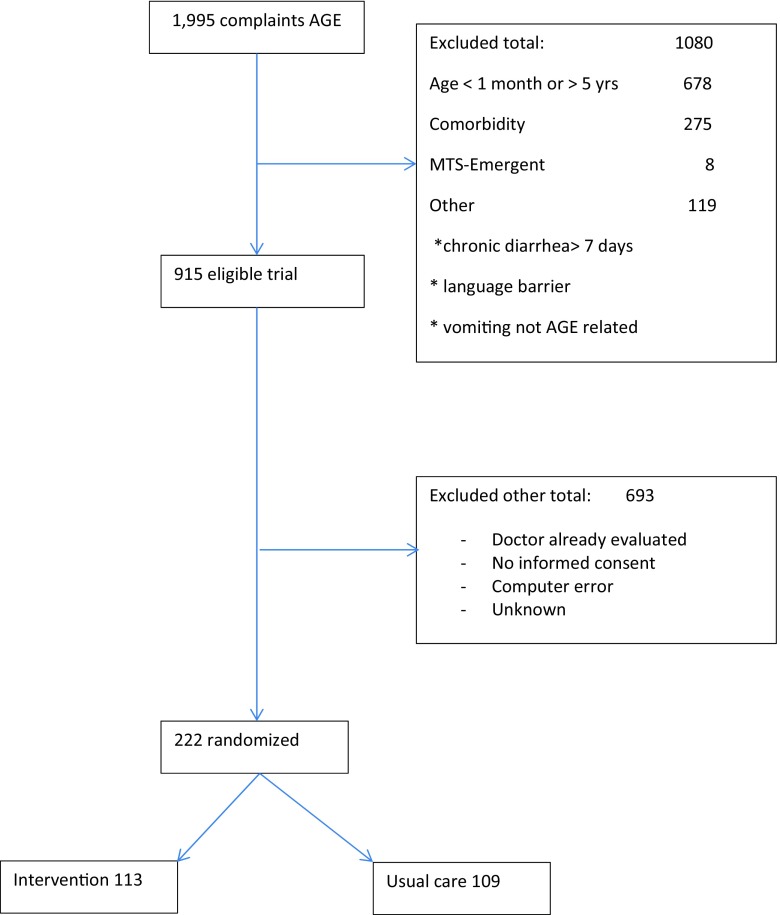
Clinical decision support system

The clinical dehydration scale and current guidelines on treatment of AGE were incorporated in an electronic, easily accessible clinical decision support system, available at each desktop at the ED [10] (Fig. 1) [1, 13, 17, 25]. At the time, anti-emetics were not a part of our national guidelines, as evidence on the use of ant-emetics, as well as on safety of ondansetron, was lacking. Therefore, we did not incorporate it in our electronic clinical decision support system.

Through the clinical decision support system, structured data were collected by the nurses on clinical signs and symptoms of all included patients. As the actual intervention included a treatment advice, most important difference with the usual care concerned a standardized amount of ORS for every dehydration level.

If randomized to the intervention, the decision support system generated a guideline-based rehydration advice corresponding to the level of dehydration of the patient (Fig. 1). The nurse generated the rehydration advice by the clinical decision support system and started the rehydration. Children with mild or moderate dehydration received orally ORS 15 ml/kg/h. Children with signs of moderate dehydration and/ or persistent vomiting received 80 ml/kg ORS per nasogastric tube in 3 h. Children without clinical signs of dehydration also started treatment to prevent dehydration and to assess tolerability of oral fluids, whilst assessing volume of ongoing losses.

The randomization was computer-generated and integrated in the clinical decision support system (randomly assigned to both groups depending on even and odd seconds of the digital computer clock). All patients, irrespective of randomization, were evaluated within the time frame generated by the triage system as well as discharged after rehydration by the attending physician. All the clinical dehydration score items had to be completed in order to get a rehydration advice in the CDS. Nurses were blinded for the contribution of predictors on the risk score. If nurse were in doubt on diagnosis and/or starting treatment, they could, at any stage, overrule the advice of the intervention and consult the attending physician.

Creating an optimal environment for implementation of the decision support system, we created group lectures for nurses at the start of their shift, repeated individual briefings and reminders by posters, email and newsletters periodically. The implementation process was closely monitored and evaluated [4].

### Data collection

We prospectively collected patient characteristics, data on signs and symptoms, vital signs, diagnostic tests, presumed diagnoses, treatment, referral, and discharge in a structured electronic hospital patient record system [21] During the study period, compliance of CDS recommendations was measured and checked with digital logbook information generated by the clinical decision support system.

To ensure correct diagnosis and ruling-out the possibility a complicated disease course, and be informed about revisits, telephonic follow-up was performed in all patients with standardized questionnaires 3 days after ED-discharge.

### Outcome measures

Feasibility was measured by compliance of the nurses to the recommendations generated by the clinical decision support system. Outcome measures included length of stay (LOS, based on triage registration until the moment of ED-discharge) at the ED, the number of diagnostic tests (electrolytes, acid-base analysis), treatment and follow-up (telephonic consultation, outpatient clinic visit, ED revisit, hospitalization), and costs. In order to determine the association between level of dehydration and weight change, the ED nurse measures the weight of all included patients at triage and intended weight measurement 24 h after discharge.

### Statistical analysis

#### Power analysis

Based on previous research at our ED, inclusion of 450 children with acute vomiting/diarrhea in 24 months was expected [22] Initial power estimates were based on the number of correct diagnosed dehydrated children, based on weight change, and its associated false positives and false negatives as described in the trial register. Despite extensive efforts, we did not succeed in repetitive weight measurements in our population, due to lack of cooperation of parents to determine weight after 24 h, neither at the ED nor elsewhere. Therefore, we were forced to recalculate power on LOS at the ED. In order to detect a reduction of 10 min consultation time (30 min standard deviation), 99 patients had to be included in each group for reliable assessment of the actual impact on LOS with a power of 0.8 and an alpha of 0.05 (one-sided test).

#### Evaluation of the clinical decision system

Being an randomized controlled trial an intention-to-treat analysis was performed.

Feasibility was measured by comparing treatment advice generated by the clinical decision support system with the actual treatment using chi-square analysis. Outcome measures were evaluated using chi-square and Student’s *t* test. Due to failure of measurement of post ED weight, we had to delete the outcome for correct diagnosis of dehydration. A *p* value <0.05 was considered statistically significant. We used SPSS version 20.0 for Windows.

#### Cost analysis

Cost analysis was performed from the hospital perspective (Appendix) [14]. Medical costs were calculated by multiplying the volumes of health care use with unit prices. We used real unit prices when available; otherwise, charges were used as a proxy for real costs. Salary schemes were used to calculate costs per hour for each health care worker. In-hospital medical costs included costs of initiated diagnostics and treatment, length of stay at the ED, hospitalization, and revisits. Volumes of diagnostics and treatment were measured according to the computer-based hospital information system. Effects of the clinical decision support system were defined as the differences in the number of false positive and false negative errors. Because the clinical decision support system resulted in comparable patient outcomes, a cost-minimization study was required. In a sensitivity analysis, we considered variation of doctor’s time and variation in costs of diagnostic tests and therapy.

## Results

**Fig. 2:**
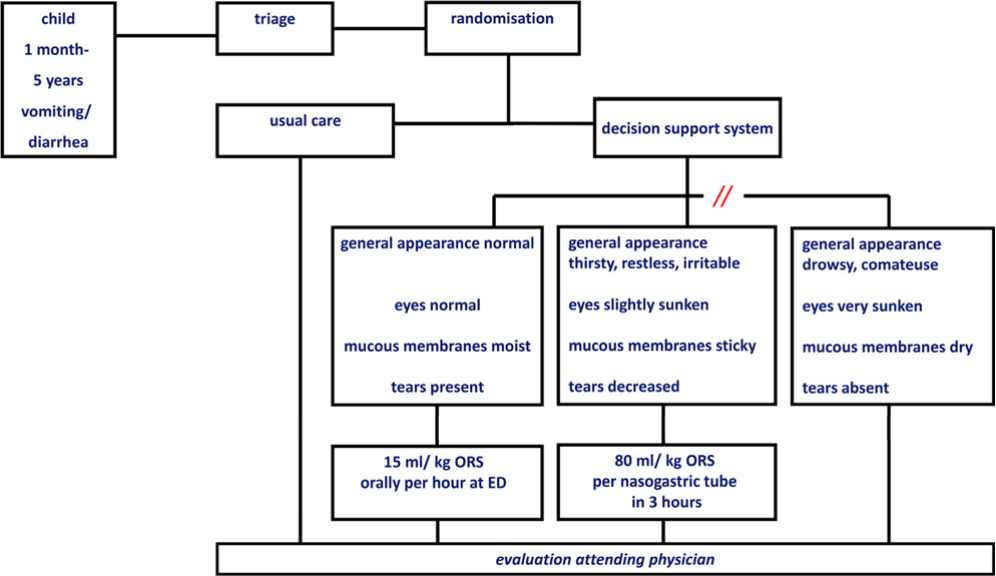
Patient flow chart

Of 915 eligible children visiting the ED with vomiting/diarrhea, 693 (75%) children were not included due to early assessment of the physician before randomization and lack of time for the nurses to obtain informed consent. We could include 222 children with informed consent in the randomized controlled trial (Fig. 2). Compared with the included population, the eligible population included more highly urgent patients according to the Manchester Triage System (indicating physicians’ evaluation within 10 min) and more patients with increased heart rate.

The intervention (*N* = 113) and the control group (*N* = 109) were comparable with respect to age (median age 1.5 years (IQR 0.9–2.4) versus 1.3 years (IQR 0.8–2.4) in the usual care group), gender, triage urgency, vital signs, and CDS-items (Table.1).

**Table 1:** Patient characteristics

	Intervention *N* = 113 (100%)^a^	Usual care *N* = 109 (100%)^a^
Age (years)^b^	1.5 (0.9–2.4)	1.3 (0.8–2.4)
Sex, male	61 (54.0)	57 (52.3)
Vital signs		
Temperature^c^ (°C)	37.6 (1)	37.7 (1)
Heart rate^c^ (beats per minute)	127 (24)	128 (21)
Respiratory rate^c^ (breaths per minute)	30 (8)	30 (8.4)
MTS urgency		
Emergent	0 (0)	1 (0.9)
Very urgent	13 (11.5)	11 (10.2)
Urgent	45 (39.8)	57 (52.8)
Standard	53 (46.9)	39 (36.1)
Non-urgent missing	2 (1.8) 0 (0.0)	0 (0) 1 (0.9)
Referral		
Own initiative	72 (63.7)	59 (54.1)
Primary care	31 (27.4)	35 (32.1)
Ambulance	4 (3.5)	1 (0.9)
Others^d^	6 (5.4)	14 (12.9)
Clinical dehydration score overall		
Mild	72 (63.7)	61 (56.0)
Moderate	41 (36.3)	47 (43.1)
Severe	0 (0)	1 (0.9)
Clinical dehydration score variables		
General appearance		
Normal	101 (89.4)	96 (88.1)
Thirsty/ restless/ irritable	11 (9.7)	13 (11.9)
Drowsy/ comatose	1 (0.9)	0 (0)
Eyes		
Normal	91 (80.5)	79 (72.5)
Slightly sunken	22 (19.5)	27 (24.8)
Very sunken	0 (0)	3 (2.8)
Mucous membranes		
Moist	101 (89.4)	91 (83.5)
Dry	12 (10.6)	18 (16.5)
Very dry	0 (0)	0 (0)
Tears		
Present	98 (86.7)	90 (82.6)
Decreased	10 (8.8)	15 (13.8)
Absent	5 (4.4)	4 (3.7)

^a^Absolute number (percentage)

^b^Median (IQR)

^c^Mean (SD)

^d^Others include secondary care and after telephone contact

### Compliance to the clinical decision support system

In the intervention group, 72/113 patients (64%) were mildly and 41/113 (36%) moderately dehydrated children, compared with 61/109 (56%) and 47/109 ( 43%) in the control group. In the intervention group, 4/88 (4.5%) children received oral rehydration solution (ORS) via a nasogastric tube. These four children refused to drink ORS (Table 2). Twelve of 25 (48.0%) children assigned to nasogastric tube rehydration by the clinical decision support system, drank the ORS themselves instead. Compliance of the nurse to the treatment advice occurred in 97/113 patients (86%; CI 95% 0.78–0.92).

**Table 2:** Outcome measures

	Intervention *N* = 113 (100%)^a^	Usual care *N* = 109 (100%)^a^	*p* value
Compliance			
Advice oral rehydration	88	Not applicable	
Compliance	84		
Non-compliance^b^	4		
Advice nasogastric tube rehydration	25		
Compliance	13		
Non- compliance^c^	12		
Patient consultation time			
Time spent at the ED (min)^d^	136 (98–206)	133 (92–184)	NS
Diagnostic procedures performed			NS
Electrolytes Acid-base balance	15 (13.3) 13 (11.5)	23 (21.1) 16 (14.7)	
Treatment procedures performed			0.01
ORS oral	73 (64.6)	57 (52.3)	0.04
ORS nasogastric tube	17 (15.0)	9 (8.3)	NS
Intravenous rehydration	0 (0.0)	2 (1.8)	NS
Liquid other	18 (15.9)	30 (27.5)	0.02
Unknown	5 (4.4)	11 (10.1)	NS
Follow-up			NS
No	57 (50.4)	59 (54.1)	
Hospitalization	10 (8.8)	7 (6.4)	
Outpatient clinic	25 (22.1)	26 (23.9)	
Telephonic consultation	21 (18.6)	15 (13.8)	
Other	0 (0.0)	2 (1.8)	
Revisits	30 (26.5)	20 (27.5)	
Hospitalization after revisit	1 (0.9)	4 (3.7)	

^a^Absolute number (percentage)

^b^Nasogastric tube by nurse in patient with oral ORS advice

^c^Oral rehydration in patient with nasogastric tube rehydration advice

^d^Median (IQR)

There were significant differences in rehydration treatment between the intervention group and the usual care group (*p* 0.01). In the intervention group, 90/113 (80%) patients received ORS, compared with 66/109 (61%) in the usual care group, fewer patients received other liquids instead of ORS ( 18/113 (16%) vs 30/109 (28%) in the usual care group), and no patients received intravenous rehydration compared with 2/109(1.8%) patients in the usual care group. In the intervention group, 10/113 (8.8%) patients were hospitalized compared with 7/109 (6.4%) in the usual care group (*p* 0.47). After discharge from the ED, 30/113 (26.5%) of the intervention group revisited the ED; one (0.9%) of these patients was hospitalized. In the usual care group, 30/109 (27.5%) revisited the ED; 4/109 (3.7%) patients were hospitalized.

### Impact of the clinical decision support system

We did not find differences between the intervention and the control group for LOS at the ED (Table 2). We observed a non-significant trend in reduction of laboratory tests, which were decreased with 50%. The number of revisits or hospitalization did not differ. All parents of children were contacted for follow-up, with a median follow-up time of 72 h. We did not observe adverse events.

### Costs

In the cost minimization study, no differences in costs in the intervention group compared with the usual care group were detected (Table 3). The differences in patient outcome between the intervention group and the usual care group consisted of more rehydration by nasogastric tube, without adverse events, and were therefore not regarded as long-term effect. Because nasogastric tubes and diagnostic tests only account for a very small part of (health care) costs, differences did not significantly influence total costs. A cost-minimization study showed comparable costs for the intervention group and the usual care group: mean costs per patient were 346 euro in the intervention group and 350 euro in the usual care group. Total (once-only) costs for development and implementation of the clinical decision support system amounted to 7000 euro/US dollar 7770 (EUR/USD 1.11; currency rate dated 8th Nov 2016).

**Table 3:** Cost-analysis (Euro)

		Intervention (*N* = 113)		Usual care (*N* = 109)	
	Cost price	Volume	Costs	Volume	Costs
CDSS					
Development (h)	36	144	5184	–	–
Implementation					
Researcher (h)	54	4	216	–	–
Nurse (number × h)	40	20 × 2	1600	–	–
Total costs CDSS implementation			7000		
ED visit					
Physician (h × visit number)	68	0.33 × 113	2535	0.33 × 109	2445
Nurse (h × visit number)	40	0.5 × 113	2260	0.5 × 109	2180
Hospital costs	114	113	12,882	109	12,426
Diagnostics					
Electrolytes	3.8	15	57	23	87
Acid-base balance	5.10	13	66	16	82
Treatment					
Unknown		6		11	
ORS portion	0.3	73	22	57	17
Liquid other	NA	18	NA	30	NA
ORS nasogastric tube	28.2	17	479	9	254
IV rehydration	5.0	0	5	2	10
Follow-up/hospitalization					
Hospitalization patients × (LOS days)	575	10 × 2	11,500	7 × 2	8050
Outpatient clinic	129	25	3225	26	3354
Telephonic follow-up	20	21	420	15	300
Costs of missed diagnoses/adverse events					
Revisit ED	144	30	4464	30	4320
Admission after revisit (LOS days × patients)	575	2 × 1	1150	2 × 4	4600
Mean costs per patient (including CDSS)			408		350
Mean costs per patient (without CDSS)			346		350

Currency rate EUR/USD 1.11; 8th Nov 2016

*LOS* length of stay

Considering a reduction of 50% doctor’s consultation time, sensitivity analysis showed a reduction of costs by 3% (12 euro/13.2 USD). Further sensitivity analyses were not performed due to relatively low impact of diagnostic and treatment costs compared with total costs.

## Discussion

We observed good compliance to the recommendations of the clinical decision support system for early rehydration in children with AGE by ED nurses and a significant increase in appropriate use of ORS compared to usual care. We did not observe adverse events. However, despite a stricter rehydration policy, we did not observe more successful rehydration in this mildly dehydrated study population, as expressed in (lack of) differences in revisits, hospitalization rates or costs.

Adherence to the treatment advice generated by the intervention was good; however, compliance to use the application of the clinical decision support system can be influenced by several factors, such as crowding hours and increased time needed to use a clinical decision support system. In our study, we indeed observed that, during crowded ED hours, the nurses did not complete all clinical dehydration score items, a prerequisite for including patients. This may point out the limitations of the implementation process. Notably, we noticed reduced inclusion of children with more severe dehydration. The substantial loss of eligible patients, and in particular those with more severe dehydration, may reflect problems of performing research and obtaining informed consent in emergency care settings as was noted by others previously [16]. Given the randomized controlled character of our trial, we believe our results remain valid. Non-included patients, however, might affect generalization of our results to more severe dehydrated children. Therefore, extrapolating our results to all dehydrated children with signs of dehydration should be done with care. The high compliance to recommendations on treatment of the clinical decision support system in the included population is encouraging for further implementation. Factors contributing to this high compliance can be explained: first, our nursing staff consists of trained pediatric nurses, who who were skilled and experienced in the clinical assessment of dehydrated children. Second, an implementation program was used [12] Third, treatment recommendations of the clinical decision support system were based on a pre-existing rehydration protocol, with which the medical staff was already familiar. Last, the clinical decision support system was designed for easy use with readily available standardized clinical items, and was accessible from every computer at the ED [3, 5].

According to the rehydration treatment guidelines underlying the decision support system, oral rehydration with ORS is recommended and biochemical testing is only indicated in severely dehydrated children. In the study population of mildly dehydrated children, the clinical decision support system showed a trend to fewer diagnostic tests and more frequent use of standardized amounts of ORS.

We did not find a beneficial effect on costs. A major reason is our low hospitalization rate, as hospitalization dominates the costs in AGE management. Nurse-guided patient assessment and treatment in the intervention group suggest that patient flow can be managed more efficiently. Sensitivity analysis on the reduction of doctor’s consultation time by 50% showed a reduction of costs by 3% (12 euro). Although this estimation is hypothetical, this might imply lower costs due to a reallocation of tasks as already described in emergency medicine, HIV care, and mental health care [2, 6, 7, 9].

### Strength and limitations

The main strength of this study is a randomized clinical trial on the impact of a clinical decision support system in pediatric emergency care. Impact analysis completes the final step in the translation process of clinical decision rules to routine practice [20]. We applied the decision support system in an electronic environment enabling easy access in routine practice and easy adaptation by other EDs.

One of our limitations is the absence of inclusion criteria based on strict signs and symptoms. Rather, we aimed for a pragmatic approach, including children with vomiting and/or diarrhea in the absence of another clear infectious focus, instead of using a strict definition based on signs and symptoms [13]. Secondly, despite our best efforts, we could not recruit the calculated number of patients. During the study period, we observed no epidemic of acute gastroenteritis. Also, we were confronted with higher than anticipated exclusion rates for serious co-morbidity as well as a high number of non-included eligible patients. Hence, we abandoned our envisioned primary endpoint and recalculated power for length of stay at the ED (LOS). One could argue that our main outcome measure LOS is subject to influence of other factors, such as admission method, discharge destination, provider (hospital) type, and specialty (acute vs non-acute). Especially, discharge destination and admission methods appeared important influencing factors [15]. As we observed hospitalization in only resp. 10 (8.8%) and 7 (6.4%) of all patients and all other patients are discharged for further treatment at home directly after finishing rehydration treatment, we think LOS was a valid outcome in our study. We expect that the presence of dedicated research personnel at the ED would have improved the whole research process, but especially the inclusion of eligible patients during crowding at the ED. Third, we could not evaluate impact on diagnosis due to failure of measurement of post ED weight. Fourth, the limited impact on (secondary) process outcome measures may be caused by the population of rather mildly dehydrated children, evaluated at a relative low-volume university hospital, and the high experienced medical and nursing staff in evaluating children. Although we proved a significant increase in appropriate use of ORS in our study, we expect larger impact also on process outcome measures in more severely dehydrated patients, or in settings with high volume or less experienced personnel.

Finally, 12 out of 25 children (48%) assigned to nasogastric route in the intervention group drank their ORS and therefore did not receive nasogastric tube. The decision support system may induce excessive use of nasogastric route. This result highlights the moderate validity of the clinical decision support system and the risks associated with the indiscriminate use of the system. The clinical decision support system must therefore be considered only as an additional tool and should not replace common sense.

## Conclusion

Implementation of a nurse-guided clinical decision support system on rehydration treatment in children with AGE showed high compliance and an increase standardized use of ORS, without differences the number of diagnostic tests, LOS, revisits, and hospitalization or costs.

## Abbreviations

AGE: Acute gastroenteritis
CDS: Clinical dehydration Scale
ED: Emergency department
LOS: Length of stay
ORS: Oral rehydration solution
